# A systematic review on the effect of sweeteners on glycemic response and clinically relevant outcomes

**DOI:** 10.1186/1741-7015-9-123

**Published:** 2011-11-17

**Authors:** Natasha Wiebe, Raj Padwal, Catherine Field, Seth Marks, Rene Jacobs, Marcello Tonelli

**Affiliations:** 1 Department of Medicine, 13-103 Clinical Sciences Building, University of Alberta, Edmonton, Alberta, T6G 2G3 Canada; 2 Department of Agricultural, Food & Nutritional Science, 410 Agriculture/Forestry Centre, University of Alberta, Edmonton, Alberta, T6G 2P5 Canada; 3 Department of Pediatrics, 8213 Aberhart Centre, University of Alberta, Edmonton, Alberta, T6G 2J3 Canada

## Abstract

**Background:**

The major metabolic complications of obesity and type 2 diabetes may be prevented and managed with dietary modification. The use of sweeteners that provide little or no calories may help to achieve this objective.

**Methods:**

We did a systematic review and network meta-analysis of the comparative effectiveness of sweetener additives using Bayesian techniques. MEDLINE, EMBASE, CENTRAL and CAB Global were searched to January 2011. Randomized trials comparing sweeteners in obese, diabetic, and healthy populations were selected. Outcomes of interest included weight change, energy intake, lipids, glycated hemoglobin, markers of insulin resistance and glycemic response. Evidence-based items potentially indicating risk of bias were assessed.

**Results:**

Of 3,666 citations, we identified 53 eligible randomized controlled trials with 1,126 participants. In diabetic participants, fructose reduced 2-hour blood glucose concentrations by 4.81 mmol/L (95% CI 3.29, 6.34) compared to glucose. Two-hour blood glucose concentration data comparing hypocaloric sweeteners to sucrose or high fructose corn syrup were inconclusive. Based on two ≤10-week trials, we found that non-caloric sweeteners reduced energy intake compared to the sucrose groups by approximately 250-500 kcal/day (95% CI 153, 806). One trial found that participants in the non-caloric sweetener group had a decrease in body mass index compared to an increase in body mass index in the sucrose group (-0.40 vs 0.50 kg/m^2^, and -1.00 vs 1.60 kg/m^2^, respectively). No randomized controlled trials showed that high fructose corn syrup or fructose increased levels of cholesterol relative to other sweeteners.

**Conclusions:**

Considering the public health importance of obesity and its consequences; the clearly relevant role of diet in the pathogenesis and maintenance of obesity; and the billions of dollars spent on non-caloric sweeteners, little high-quality clinical research has been done. Studies are needed to determine the role of hypocaloric sweeteners in a wider population health strategy to prevent, reduce and manage obesity and its consequences.

## Background

Non-caloric sweeteners have been available commercially since the late 1800s [1] and their use in food products and as table-top sweeteners is increasing - perhaps due in part to aggressive marketing promoting their capacity to induce weight loss and weight maintenance [2,3]. In 2007, non-caloric and/or high-intensity sweeteners accounted for 16% of the US sweetener market (approximately 0.5 billion USD [4]) and projected sales of these products are expected to exceed one billion USD by 2014 [5].

Sugar alcohols can also be used as sweetener additives and provide less calories per gram than saccharides (sugars). However because sugar alcohols cause gastrointestinal symptoms in some individuals due to incomplete absorption in the small intestine, they may be used less frequently than saccharides. A variety of different saccharides is commonly used to sweeten foods, such as sucrose, fructose, glucose, maltose, isomaltulose, and fructooligosaccharide (FOS). FOS has half the calories per gram than sucrose, fructose, or glucose. Most recently, fructose (a highly commercially used sweetener used in combination with glucose as high fructose corn syrup (HFCS)) has been controversially linked with hypertriglyceridemia [6].

The effects of different sweeteners on clinically relevant outcomes such as weight management, blood glucose and lipids have been incompletely studied. The main metabolic complications of obesity and type 2 diabetes may be prevented and managed in full or in part with dietary modification, including the use of sweeteners that provide little or no calories (hypocaloric sweeteners) [7-10].

This review systematically summarizes the available randomized trial evidence to determine the comparative effectiveness of sweetener additives (non-caloric, sugar alcohols, and saccharides; Table 1) in food.

**Table 1:** Description of sweeteners

Sweetener Commercial products	Nutritive, kcal/g	**Sweetness intensity**, relative to sucrose
Non-caloric		
Acesulfame-K [83-85] Sunett^®^	0	200
Aspartame [83-86] Equal^®^, NutraSweet^®^	4	180
Cyclamate [83-85]	0	30-50
Saccharin [83-85] Sweet'N Low^®^, Sugar Twin^®^, Hermesetas^®^	0	300-500
Sucralose [83-85] Splenda^®a^	0	600
Sugar alcohol		
Hydrogenated starch hydrolysate (HSH) [85]	≤3	0.4-0.9
Lycasin [87]	2.4	0.75
Maltitol [85]	3	0.9
Sorbitol [85,86]	2.6	0.6
Saccharide		
Fructooligosaccharides (FOS) [88]	2	0.3-0.6
Fructose [85,86,89]	4	1-2
Glucose [86,89]	≥ 4	0.5-1
High fructose corn syrup (HFCS) [85,89] Varieties: HFCS 55, HFCS 42, HFCS 90	≥ 4	~1
Honey [90]	≥ 4	1-1.5
Isomaltulose [88,91] Palatinose	4	0.5
Maltose [86,89]	≥ 4	0.5
Sucromalt [88]	4	0.7
Sucrose [86,89]	4	1 (reference)
Tagatose [83]	1.5	0.9
Trehalose [92]	4	0.45

^a^Splenda also contains maltodextrin and sometimes dextrose which are both nutritive

## Methods

This systematic review was conducted and reported according to guidelines [11].

### Data sources and searches

We did a comprehensive search designed by a MLIS-trained librarian to identify all randomized controlled trials (RCTs) comparing sweeteners in generally healthy, overweight/obese and/or diabetic participants. We included only trials published in English as full peer-reviewed manuscripts. MEDLINE (1950 to January 13, 2011), EMBASE (1980 to January 13, 2011), CENTRAL (January 13, 2011), and CAB (January 13, 2011) were searched. No existing systematic reviews were found. The specific strategies used are provided in Additional File 1. The citations and abstracts were screened by two reviewers to identify pertinent trials. Any study considered potentially relevant by one or both reviewers was retrieved for further consideration.

### Study selection

We considered non-caloric sweeteners to include high-intensity caloric sweeteners that are functionally non-caloric simply due to extremely low doses (for example, aspartame). Each potentially relevant study was independently assessed by two reviewers for inclusion in the review using predetermined eligibility criteria. Disagreements were resolved by consultation with a third party. Trials with healthy, overweight/obese, and/or diabetic adult (≥ 16 years old) participants meeting the following criteria were eligible for inclusion: parallel or crossover RCTs; weight change, energy intake, lipids, glycated hemoglobin (HbA1C), or insulin resistance were reported; had at least two groups comparing different sweeteners (for example, glucose, fructose, sucrose, other saccharides, sugar alcohols, non-caloric sweeteners: aspartame, saccharin, stevioside, sucralose); and where follow-up was at least one week in duration (see the Box in Additional File 1 for study selection summary). RCTs measuring 2-hour blood (serum or plasma) glucose responses in similar populations without the follow-up requirement were also reviewed. All outcomes selected for study (including weight change) are reversible and thus (providing that order was randomly assigned), a cross-over design should be appropriate. Trials with less than ten participants per group were excluded to improve the efficiency of the work without an appreciable loss of power, and with the possible elimination of some small study bias. Trials aimed at evaluating exercise performance or memory enhancement were excluded. Trials with placebo controls were also excluded as we aimed to investigate comparative effectiveness of different sweeteners, as opposed to exploring the implications of avoiding sweeteners altogether.

### Data extraction and quality assessment

A standardized data extraction method was performed by a single reviewer. A second reviewer checked the extracted data for accuracy. The following properties of each trial were recorded in a database: trial characteristics (country, design, sample size, duration of follow-up); participants (age, gender, co-morbidity (obesity, diabetes mellitus - type 1 and 2), baseline body mass index (BMI), diabetic therapy (insulin, oral antihyperglycemic agents, diet, and so on); sweetener characteristics (type, quantity, schedule); diet (that is, daily caloric content by macronutrient/fiber content); and outcomes. Outcomes included weight change (absolute, BMI), energy intake, lipid measures (total cholesterol, triglycerides, high density lipoprotein (HDL), low density lipoprotein (LDL)), HbA1C, insulin resistance (for example, Homeostatic Model Assessment (HOMA) index), and 2-hour blood glucose (with or without meals).

Risk of bias was assessed using items known to be associated with the magnitude of results (that is, method of randomization, double-blinding, description of withdrawals/dropouts, and allocation concealment) [12,13]. Source of funding was also extracted given its potential to introduce bias [14].

### Data synthesis and analysis

Data were analyzed using Stata 11.1 (http://www.stata.com). Missing standard deviations (SDs) were imputed using the maximum value reported in any included study [15]. Missing correlations for change from baseline and for differences between crossover trial periods were assigned a value of 0.63, the maximum reported value in the included studies. Changes from baseline means were used in place of final means in parallel randomized trials. For weight change, the baseline value prior to the immediate period was used. The mean difference (MD) was used to summarize outcomes. Due to expected diversity between studies, we decided *a priori *to combine results using a random effects model (Stata command: metan). Additionally we planned to examine the association between certain variables (population, dose, diet, age, gender, and bias criteria) and the effect of specific sweeteners on outcomes, and publication bias with weighted regression [16], however the available comparisons were too sparse to pool trials with outcomes of one week or less. For 2-hour responses, we pooled comparisons by type of sweetener and ordered the matrix tables by expected order of efficacy [17] (that is, non-caloric sweeteners, sugar alcohols, other saccharides, fructose, sucrose, and glucose). Statistical heterogeneity was quantified using the τ^2 ^statistic (between-study variance) [18]. Furthermore, we explored comparative effectiveness of sweeteners on 2-hour responses using network meta-analysis [19] (specifically, Markov chain Monte Carlo [MCMC] methods within a Bayesian framework) in WinBugs (http://www.mrc-bsu.cam.ac.uk/bugs; code was obtained from Ades *et al*. [20]). Network analysis extends meta-analysis from simply pooling directly compared treatments (direct evidence) to pooling data from studies not directly compared but linked via one or more common comparators (indirect evidence) by assuming consistency of the evidence [19]. Therefore, this technique facilitates the comparison of any two sweeteners not directly compared in any one study. We used non-informative prior distributions: uniform for the between-study variance (range 0 to 20) and Gaussian for the other parameters (mean 0 and variance 10,000). All chains were run for 10,000 iterations after 1,000 burn-in iterations. Convergence of the MCMC algorithm was assessed using autocorrelation plots. By-population results were generated. Inconsistency in the network (disagreement between direct and mixed evidence) was measured using back-calculations [21]. Ninety-five present Bayesian credible intervals are reported.

## Results

### Quantity of research available

**Figure 1:**
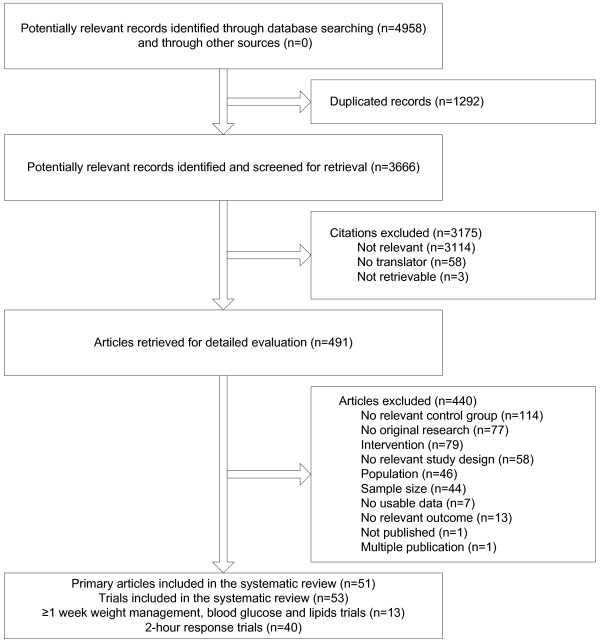
**Flow diagram of trials considered for inclusion**. This flow diagram depicts each step in the process of trial selection.

The searches identified 3,666 unique records with no trials found outside the main literature searches. After initial screening, 491 articles were retrieved for detailed evaluation (Figure 1) and of these 440 articles were excluded resulting in 53 trials (from 51 publications) that met the selection criteria. Disagreements about the inclusion of studies occurred in 11% of the articles (kappa = 0.71). Fourteen were ultimately included. The remaining were excluded for the following reasons: thirteen with no relevant control group, nine with no relevant population, five with no relevant intervention group, four due to study design, four for small sample size, and one for no usable data. The sweeteners studied in eligible trials are described in Table 1.

### Characteristics of 2-hour response trials

Of the forty included trials with 2-hour response data (703 participants; Table 2), three trials compared a non-caloric sweetener (aspartame [22,23], or sucralose [24]) to a saccharide (fructose [24] or sucrose [22,23]); one trial compared a non-caloric to another non-caloric (aspartame versus saccharin [25]); four trials compared a sugar alcohol or a malt containing a sugar alcohol (sorbitol [26], xylitol [26,27], maltitol [26], Lycasin [26,28], or a hydrogenated starch hydrolysate (HSH) [29]) to a saccharide (glucose [26,28,29] or sucrose [27]); and thirty-two trials compared a saccharide to another saccharide (glucose [30-51], fructose [31-34,36,38-41,44-47,49,50], mixtures of glucose and fructose [that is, sucrose [34,37,39,42,43,48-57], HFCS [42,54,55,58], honey [48,57,59], glucose/fructose equivalent honey [59]], isomaltulose [52,56], maltose [53], sucromalt [58], trehalose [30], or a mixture of trehalose and fructose [30]). Approximately half of the doses for saccharides were less than the 60 g/day recommended for diabetic patients on a 2,000 kcal diet; the remainder exceeded 60 g/day (typically 75 g). All of the doses for sugar alcohols exceeded the 10 g/day recommendation (range 20 to 50 g), which is aimed at limiting gastrointestinal symptoms. None of the four non-caloric sweetener groups were above Acceptable Daily Intake (ADI) values.

**Table 2:** Description of included 2-hour response randomized trials

**Author**, **Year**, Country	**Population**, Mean BMI (kg/m^2^)	Sweetener 1: **Type**, Quantity	Sweetener 2: **Type**, Quantity	Medium	**Study design**, **Follow-up**, Sample size	**Mean age (y)**, % female	**Allocation concealment**, **Jadad score**, Funding
Non-caloric vs Saccharide							
Gonzalez-Ortiz [24] 2009 Mexico	General 23	Sucralose (Enterex Diabetic^®^) 45% once	Mixture^b^ (Glucerna SR^®^) 50% once	Drink	xRCT 2 1d periods (3d wo) 14	22 64	Unclear 2 -
Prat-Larquemin [22] 2000 France	General 20	Aspartame 0.27 g once	Sucrose 90 g once	Cheese	xRCT 2 1d periods (1wk wo) 24	23 0	Unclear 3 Mixed^e^
Melchior [23] 1991 France	General 21	Aspartame 80mg once	Sucrose 50 g once	Drink	xRCT 2 1d periods 10	22 70	Unclear 1 Public
Non-caloric vs Non-caloric							
Horwitz [25] 1988 US	55% General/45% DM2 < 25	Aspartame 400 mg once	Saccharin 135 mg once	Unsweetened drink	xRCT 2 1d periods (1wk wo) 22	41 77	Unclear 1 Private
Sugar Alcohol vs Saccharide							
Rizkalla [28] 2002 France	50% General 25 50% DM2 27	Lycasin 50 g once	Glucose 50 g once	None	xRCT 2 1d periods (1wk wo) 12	40 0	Unclear 2 Private
Nguyen [26] 1993 France	General -	Lycasin 20 g once Sorbitol 20 g once Xylitol 20 g once Maltitol 20 g once	Glucose 20 g once	None	xRCT 5 1d periods (1wk wo) 10	33 50	Unclear 1 Private
Wheeler [29] 1990 US	33% General 24^f^ 33% DM2 32 33% DM1 23	HSH6075 50 g once HSH5875 50 g once	Glucose 50 g once	None	xRCT 3 1d periods 18	47 50	Unclear 2 Mixed
Hassinger [27] 1981 Germany	DM1 -	Xylitol 30 g once	Sucrose 30 g once	Meal	xRCT 2 1d periods 14	29 -	Unclear 1 Private
Saccharide vs Saccharide							
Maki [30] 2009 US	Obese 35	Trehalose 75 g once	Trehalose/Fructose 75 g once Glucose 75 g once	None	xRCT 3 1d periods 21	50 0	Unclear 3 Private
Teff [31] 2009 US	Obese 35	Fructose 30%	Glucose 30%	Meals & Drinks	xRCT 2 2d periods (1mo wo) 17	33 47	Unclear 1 Public
Van Can [52] 2009 Netherlands	Overweight 28	Isomaltulose 75 g once	Sucrose 75 g once	None	xRCT 2 1d periods (1wk wo) 10	31 20	Unclear 1 Private
Grysman [58] A 2008 Canada	General 24	Sucromalt 50 g once	HFCS42 50 g once	None	xRCT 2 1d periods 10	28 30	Unclear 2 Private
Grysman [58] B 2008 Canada	General 24	Sucromalt 80 g once	HFCS42 80 g once	None	xRCT 2 1d periods 10	25 50	Unclear 2 Private
Grysman [58] C 2008 Canada	General 24	Sucromalt 50 g once	HFCS42 50 g once Glucose 50 g once	Meal	xRCT 3 1d periods 20	37 60	Unclear 2 Private
Munstedt [59] 2008 Germany	General 23	Glucose/Fructose^a^ 155 g once	Honey 221 g once	None	xRCT 2 1d periods (1wk wo) 10	28 0	Unclear 2 -
Stanhope [54] 2008 US	General 25	HFCS55 25% thrice	Sucrose 25% thrice	3 Meals	xRCT 2 1d periods (1mo wo) 34	35 47	Unclear 1 Mixed
Bowen [32] 2007 Australia	Overweight/ Obese 33	Fructose 50 g once	Glucose 50 g once	Drink	xRCT 2 1d periods (7d wo) 28	57 0	Unclear 4 Mixed
Chong [33] 2007 UK	General 25	Fructose 0.75 g/kg once	Glucose 0.75 g/kg once	Drink	xRCT 2 1d periods (6wk wo) 14	43 43	Unclear 1 Mixed
Melanson [55] 2007 US	General 22	HFCS55 30% TID	Sucrose 30% TID	3 Meals	xRCT 2 2d periods (6wk wo) 30	33 100	Unclear 2 Private
Visvanathan [34] 2005 Australia	General 26	Sucrose 50 g once Fructose 50 g once	Glucose 50 g once	None	xRCT 3 1d periods (3d wo) 10	72 60	Unclear 1 Public
Teff [35] 2004 US	General 23	Fructose 30% TID	Glucose 30% TID	3 Meals	xRCT 2 2d periods (1mo wo) 12	25 100	Unclear 1 Mixed
Qin [53] 2003 Japan	General 23	Maltose 75 g QIDx4h	Sucrose 75 g QIDx4h	None	xRCT 2 1d periods (1wk wo) 10	22 0	Unclear 1 -
Vozzo [36] 2002 Australia	50% IGT/50% DM2 31	Fructose 75 g once	Glucose 75 g once	None	xRCT 2 1d periods (5d wo) 20	56 40	Unclear 1 Mixed
Spiller [37] 1998 US	General -	Sucrose 90 g once	Glucose 90 g once	None	xRCT 2 1d periods (1wk wo) 10	29 50	Unclear 1 Private
Stewart [38] 1997 Canada	General 20-27	Fructose 30 g SID	Glucose 33.5 g SID	Meal	xRCT 2 1d periods 13	25 0	Unclear 1 Private
Blaak [39] 1996 Netherlands	General -	Sucrose 75 g once Fructose 75 g once	Glucose 75 g once	None	xRCT 3 1d periods (1wk wo) 10	28 0	Unclear 1 Mixed
Fukagawa [40] 1995 US	General -	Fructose 75 g once^d^	Glucose 75 g once	None	xRCT 2 1d periods 16	47 38	Unclear 1 Public
Schwarz [41] 1992 Switzerland	43% General 21 57% Overweight 30	Fructose 75 g once	Glucose 75 g once	Meal	xRCT 2 1d periods (4d wo) 23	25 100	Unclear 1 Mixed
Bukar [42] 1990 US	DM2 -	HFCS 27 g (12.2 g Fructose/14.8 g Glucose) once Sucrose 33.5 g once	Glucose 50 g once	HFCS: Tofu frozen dessert Sucrose: Ice cream Glucose: None	xRCT 3 1d periods (2d wo) 12	51 50	Unclear 1 -
Georgakopoulos [43] 1990 Greece	General -	Sucrose 20 g once	Glucose 20 g once	None	xRCT 2 1d periods (3d wo) 17	Range 25-40 29	Unclear 1 -
Kawai [56] 1989 Japan	50% General 20 50% DM2 23	Isomaltulose 50 g once	Sucrose 50 g once	None	xRCT 2 1d periods (2d wo) 20	39 30	Unclear 1 Mixed
Schwarz [44] 1989 Switzerland	General 21	Fructose 75 g once	Glucose 75 g once	Drink	xRCT 2 1d periods (4d wo) 20	23 50	Unclear 1 Private
Simonson [45] 1988 Switzerland	DM2/General/Obese^f^ -	Fructose 75 g once	Glucose 75 g once	None	xRCT 2 1d periods (1wk wo) 37	53 51	Unclear 1 Mixed
Jansen [46] 1987 Netherlands	General -	Fructose 75 g once	Glucose 75 g once	None	xRCT 2 1d periods (1wk wo) 20	52 50	Unclear 1 Public
Tappy [47] 1986 Switzerland	General -	Fructose 75 g once	Glucose 75 g once	None	xRCT 2 1d periods (2d wo) 10	27^c^ 65^c^	Unclear 1 Mixed
Erkelens [57] 1985 Netherlands	33% General^f^/33% DM2/17% DM1/17% insulin infusion DM1 -	Honey (22% Glucose/26% Fructose) SID	Sucrose 49% SID	White bread & Cheese	xRCT 2 1d periods (2d wo) 24	47 46	Unclear 1 Mixed
Samanta [48] 1985 UK	46% General/31% DM1/23% DM2 -	Honey 26 g once Sucrose 26 g once	Glucose 26 g once	None	xRCT 3 1d periods 26	40 -	Unclear 1 -
Bantle [49] 1983 US	31% General/38% DM1/31% DM2 -	Sucrose 42 g once Fructose 42 g once	Glucose 42 g once	Meal	xRCT 3 1d periods 32	41 56	Unclear 2 Mixed
Crapo [50] 1982 US	38% General/23% IGT^f^/38% DM2 -	Sucrose 63 g once Sucrose 52 g once Fructose 63 g once Fructose 52 g once	Glucose 69.9 g once	Sucrose & Fructose 63 g: cake Sucrose & Fructose 52 g: ice cream None	xRCT 5 1d periods (1d wo) 26	43 42	Unclear 1 Mixed
Mann [51] 1971 South Africa	General -	Sucrose 60 g once	Glucose 60 g once	Meal	xRCT 2 1d periods (2d wo) 19	Range 20-58 0	Unclear 1 Public

DM1, type 1 diabetes mellitus; DM2, type 2 diabetes mellitus; IGT, impaired glucose tolerance; UK, United Kingdom; US, United States; HFCS, high fructose corn syrup; FOS, fructooligosaccharide; SID, once a day; BID, twice a day; TID, three times a day; QID, four times a day; carb, carbohydrate; xRCT, randomized crossover trial; RCT, parallel randomized controlled trial; wo, washout; max, maximum "-" means the value was not reported in the study, and not described

^a^'comparable Honey'

^b^contains isomaltulose, fructose, Sucromalt^®^ (sucrose, maltose), and FOS

^c^approximate because it includes 7 people from other studies written up in the same article

^d^factorial trial including 300 mg caffeine or 300 mg vitamin C

^e^both public and private sources of funding

^f^group of participants dropped due to low group sample size

Twelve trials included diabetic populations (range mean BMI 23 to 32 kg/m^2^) [25,27-29,36,42,45,48-50,56,57], five trials exclusively studied overweight or obese individuals (range mean BMI 28 to 35 kg/m^2^) [30,31,41,45,52], and thirty-five trials included generally healthy individuals (range mean BMI 20 to 26 kg/m^2^). Median mean age was 35 years (range 22 to 72 years) and median sex distribution was 47% women.

Sample size ranged from 10 to 37 (median 17), three studies (8%) had sample sizes ≥ 30 per group and all were randomized crossover trials. The median Jadad score was 1 (range 1 to 4); no studies reported concealing treatment allocation.

### 2-hour blood glucose response

**Figure 2:**
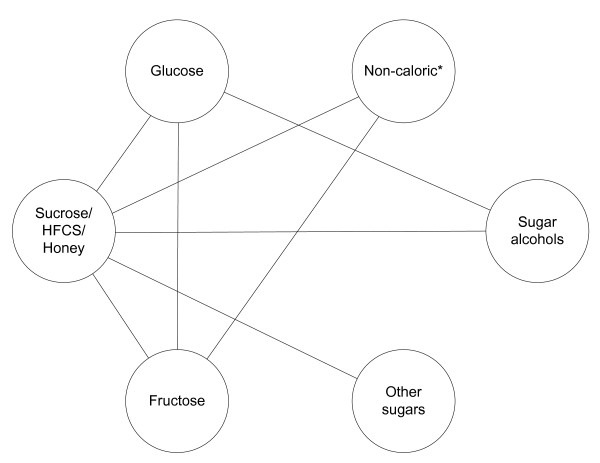
**Network: blood glucose (mmol/L) at 2 hours post-sweetener consumption and overnight fast**. HFCS, high fructose corn syrup. *non-caloric sweetener groups were unavailable in the network with diabetic participants.

Table 3 reports the results of the direct meta-analysis for all populations in the lower triangle and the mixed evidence from the Bayesian network (Figure 2) in the upper triangle. The network included 36 trials and 610 participants. The direct evidence from all nine comparisons was consistent with the mixed evidence from the network. There was large heterogeneity between trials (I^2^'s ≥ 77%) for three of seven multi-study direct evidence comparisons. Two of the heterogeneous comparisons included a variety of sweeteners (that is, multiple sugar alcohols (τ^2 ^= 9.05 (95% CI 2.94,32)), or multiple other sugars (τ^2 ^= 1.72 (0.37,1.48))) within one category. In the fructose versus glucose comparison, six trials were responsible for the heterogeneity (τ^2 ^= 1.40 (0.68,1.50)). Three [36,45,50] were subgroups of diabetic participants; they increased the magnitude of the mean difference. The other three trials [32,33,46] showed important differences prior to the 2-hour time point (data not shown) but at two hours showed little or no difference between sweeteners. The single estimate of heterogeneity (τ^2^) for the network meta-analysis was 0.65 (95% CI 0.35,1.10).

**Table 3:** Mean difference in serum glucose (mmol/L) at 2 hours post-sweetener consumption and overnight fast in all participants

**Non-** **caloric** 0.05	0.98 (-1.24,3.25) τ^2 ^=0.65 (0.35,1.10)	0.16 (-1.46,1.80) consistent	1.19 (-0.56,2.94)	0.07 (-1.45,1.61) consistent	-0.37 (-2.07,1.29)
-	**Sugar** **alcohols** 0.38	-0.83 (-2.66,1.03)	0.21 (-1.47,1.84)	-0.93 (-2.56,0.70) consistent	-1.37 (-2.96,0.18) consistent
**-0.40** **(-0.79,-0.01)** N = 1	-	**Other** **sugars** 0.01	1.03 (-0.13,2.20)	-0.09 (-1.00,0.81) consistent	-0.55 (-1.61,0.50) consistent
-	-	-	**Fructose** 0.55	**-1.12** **(-1.95,-0.27)** consistent	**-1.56** **(-2.18,-1.02)** consistent
0.30 (-1.99,2.58) N = 2 I^2 ^= 0 τ^2 ^= 0	0.41 (-2.44,3.26) N = 1	-0.28 (-1.67,1.11) N = 7 I^2 ^= 84 τ^2 ^= 1.72 (0.37,1.48)	-0.41 (-1.30,0.47) N = 9 I^2 ^= 11 τ^2 ^= 0.17 (0.58,2.41)	**Sucrose/** **HFCS/** **Honey** 0	-0.45 (-1.15,0.21) consistent
-	-2.20 (-10.46,6.05) N = 3 I^2 ^= 85 τ^2 ^= 9.05 (2.94,32.22)	0.10 (-2.46,2.66) N = 2 I^2 ^= 0 τ^2 ^= 0	**-1.40** **(-2.05,-0.74)** N = 23 I^2 ^= 77 τ^2 ^= 1.4 (0.68,1.50)	**-0.31** **(-0.53,-0.08)** N = 15 I^2 ^= 0 τ^2 ^= 0 (0,0.28)	**Glucose** 0

HFCS, high fructose corn syrup

The mixed evidence of the Bayesian network analysis are in the upper triangle and the direct evidence calculated using the REML estimate of τ^2 ^are in the lower triangle. Sweeteners are reported in the expected order of efficacy[17] (with the exception of other sugars) from the expected lowest to highest 2-hour glucose response, with the estimated probability (or rank) listed in the diagonal. Each table cell contains the mean difference (MD) with the accompanying 95% confidence intervals. In the cells with direct evidence, we also list the number of studies, the I^2 ^(percent of heterogeneity due to between-study heterogeneity) and τ^2 ^(the between-study variance). Blank cells in the lower triangle indicate that no direct evidence was available. In the cells with mixed evidence, we list whether the mixed evidence was consistent with the available direct evidence. Also, in the first cell of the mixed evidence, we list the single τ^2 ^estimate for the mixed evidence. Results are the MD of the expected higher-ranked sweeteners compared to the expected lower-ranked sweeteners (for example, MD of sugar alcohols versus sucrose is 0.41 and is in column 2, row 5 for the direct results, and is -0.93 and is in column 5, row 2 for the network analysis results). MDs less than zero favor the expected higher-ranked sweetener (smaller glucose response). For example, sugar alcohols show an increased serum glucose response by 0.41 mmol/L compared to sucrose using the direct evidence. However, sugar alcohols show a decreased serum glucose response by 0.93 mmol/L using the mixed evidence. However, since both confidence intervals include zero, neither analysis allows a confident judgment about which sweetener is preferable. Pooled evidence significant at *P *< 0.05 are presented in bold font. All nine mixed and direct results are consistent.

Reporting the mixed evidence, two comparisons: fructose versus sucrose (MD -1.12 mmol/L (-1.95,-0.27)), and fructose versus glucose (-1.56 mmol/L (-2.18,-1.02)) were statistically significant, all favoring fructose, but neither of the confidence limits excluded the possibility of non-clinically relevant differences (< 1·15 mmol/L - calculation based on a clinical important difference of 1% for HbA1C) [60]. The weighted regression test for publication bias was not significant.

In the subnetwork of 31 trials enrolling participants without diabetes (446 participants; τ^2 ^= 3.66 (1.66,7.31); Appendix Table 1 in Additional File 1), the direct evidence from all 8 comparisons was consistent with the mixed evidence from the network. The heterogeneity although reduced remained large between trials (I^2^'s ≥ 60%) in both of the remaining multi-study direct evidence comparisons. Using the mixed evidence, three comparisons: fructose versus sucrose (-0.54 mmol/L (-1.06,-0.03)), fructose versus glucose (-0.89 mmol/L (-1.21,-0.59)), and fructose versus other sugars (-0.85 mmol/L (-1.47,-0.21)) were statistically significant, all favoring fructose, but none of the confidence limits excluded the possibility of non-clinically relevant differences.

In the subnetwork of ten trials enrolling participants with diabetes (152 participants; Appendix Table 2 in Additional File 1), the direct evidence from all six comparisons was consistent with the mixed evidence from the network. Note, this network did not include non-caloric sweeteners. Because the estimate of τ^2 ^(224 (0.14,139)) did not converge, we report our findings from the direct evidence. Three direct comparisons were significant and found clinically relevant differences between agents over the entire confidence interval span: fructose versus glucose in 5 trials with 52 participants (-4·81 mmol/L (-6·34,-3·29), I^2 ^= 0%, τ^2 ^= 0 (0,7.47)), HSH versus glucose in 1 trial [29] with 12 participants (-6·19 mmol/L (-9·78,-2·60)) and isomaltulose versus sucrose in 1 trial [52] with 20 participants (-3·44 mmol/L (-5·31,-1·56)).

### Characteristics of trials studying effects on weight management, blood glucose and blood lipids

Of the 13 trials (412 participants; Table 4), 3 trials compared a non-caloric sweetener (aspartame [61], cyclamate [62], or a mixture [63]) to sucrose, and 10 trials compared a saccharide to a different saccharide (glucose [64-66], fructose [64,65,67], mixtures of glucose and fructose [that is, sucrose [66-72] or honey [69]], FOS [71-73], a mixture of isomaltulose and sucrose [68], or tagatose [70]). No trials evaluated stevioside. Seven trials did not give any daily diet recommendations; one FOS trial recommended low-fiber intake [72]; one restricted added sweeteners to the assigned sweetener [62]; three trials restricted total energy levels and composition of macronutrients (55% carbohydrate, 30% fat, 15% protein) [64,65,67]; and another restricted total energy levels and the composition to the assigned sweetener plus calcium caseinate [66]. With three exceptions [63,64,66], the doses of sweeteners were at or below current clinical practice guideline (CPG) recommendations (10% of total energy intake (for example, 60 g of sucrose in a 2000 kcal diet) although only three trials [64,65,67] restricted overall energy intake, therefore further sweetener consumption may have exceeded current recommendations. One trial [63] prescribed sweeteners (simple carbohydrates) at 25% of total energy intake - the American Diabetes Association (ADA) 2004 recommended maximum. The earliest trial [66] prescribed sweeteners at 87% of total energy intake - they were differentiating energy availability from energy content.

**Table 4:** Characteristics of included randomized trials with effects on weight management, blood glucose and blood lipids

**Author**, **Year**, Country	**Population**, Mean BMI (kg/m^2^)	Sweetener 1: **Type**, Quantity (g/d)	Sweetener 2: **Type**, Quantity (g/d)	Daily Diet (carbohydrate/ fat/protein)	**Study design**, **Follow-up**, Sample size	**Mean age (y)**, % female	**Allocation concealment**, **Jadad score**, Funding
Non-caloric versus Saccharide							
Reid [61] 2007 UK	General 23	Aspartame 3.56	Sucrose 42	Ad lib	RCT 4 wk 133	32 100	Unclear 1 Public
Raben [63] 2002 Denmark	Overweight 28	Aspartame/Acesulfame/ Cyclamate/Saccharin 0.48-0.67	Sucrose 125-175	Ad lib	RCT 10 wk 41	35 85	Unclear 1 Mixed^a^
Chantelau [62] 1985 Germany	DM1 < 25	Cyclamate 348 mg	Sucrose 24	Restricted to no other added sweeteners however sucrose-sweetened soft drinks were discouraged	xRCT 2 4wk periods 10	Range 25-43 80	Unclear 1 -
Saccharide versus Saccharide							
Okuno [68] 2010 Japan	General 23	Isomaltulose/ Sucrose 40	Sucrose 40	Ad lib	RCT 12 wk 50	53 80	Unclear 2 Private
Tudor Ngo Sock [64] 2010 Netherlands	General 19-25	Fructose 3.5 g/kg FFM	Glucose 3.5 g/kg FFM	Total and distribution of energy restricted 55/30/15%	xRCT 2 1wk periods (2-3wk wo) 11	25 0	Unclear 1 Mixed
Yaghoobi [69] 2008 Iran	Overweight/ Obese 31	Honey 70	Sucrose 70	Ad lib	RCT Max 30d 55	42 56	Unclear 1 Public
Boesch [70] 2001 Switzerland	General < 25	Tagatose 45	Sucrose 45	Ad lib	xRCT 2 28d periods (28d wo) 12	Range 21-30 0	Inadequate 2 -
Bantle [65] 2000 US	General 25	Fructose 80 (incl 17 g glucose)	Glucose 80 (incl 15 g fructose)	Total and distribution of energy restricted 55/30/15%	xRCT 2 42d periods 24	41 50	Unclear 1 Public
Luo [71] 2000 Belgium	DM2 28	FOS 20	Sucrose 20	Ad lib	xRCT 2 4wk periods (2wk wo) 10	57 40	Unclear 1 Private
Alles [73] 1999 Netherlands	DM2 28	FOS Saccharide 30	Glucose Saccharide 8	Ad lib	xRCT 2 20d periods 20	59 55	Unclear 1 Mixed
Luo [72] 1996 France	General 21	FOS 20	Sucrose 20	Low-fiber diet recommended	xRCT 2 4wk periods (2wk wo) 12	24 0	Unclear 2 Private
Bantle [67] 1986 US	50% DM1/50% DM2 -	Sucrose 23%	Fructose 21%	Total and distribution of energy restricted 55/30/15%	xRCT 2 8d periods 24	43 54	Unclear 1 Mixed
Macdonald [66] 1973 UK	General -	Sucrose 6.5 g/kg	Glucose 6.5 g/kg	Restricted to 1 g/kg calcium caseinate	xRCT 2 11d periods (2wk wo) 10	Range 20-25 40	Unclear 1 Private

UK, United Kingdom; US, United States; DM1, type 1 diabetes mellitus; DM2, type 2 diabetes mellitus; HFCS, high fructose corn syrup; FOS, fructooligosaccharide; FFM, fat free mas;, xRCT, controlled crossover trial; RCT, parallel randomized controlled trial; wo, washout; max, maximum; "-" means the value was not reported in the study

^a^Both public and private sources of funding

Four trials were in diabetic populations [62,67,71,73], seven trials were in generally healthy populations [61,64-66,68,70,72] and two trials were in overweight/obese [69] or overweight [63] populations. Mean BMI levels ranged from 21 to 31 kg/m^2^. Median mean age was 35 years and median sex distribution was 54% women.

Sample size ranged from 10 to 133 (median 20), 1 had a sample size ≥ 30 per group and duration of follow-up ranged from 1 to 12 weeks (median 4 weeks). Ten were crossover trials [62,64-67,70-73] and four were parallel trials [61,63,68,69]. Jadad scores ranged from 1 to 2 (median 1). Twelve of thirteen trials did not report whether or how treatment assignment was concealed. One used alternating assignments according to body weight [70].

### Non-caloric versus saccharide: effects on weight management, blood glucose and blood lipids

Two trials reported change in BMI (Table 5). The 4-week trial in healthy participants [61] did not find a significant loss in BMI in non-caloric sweetener recipients (-0.3 kg/m^2 ^(-1.1,0.5), 133 participants). The trial in overweight participants [63] found a significantly greater loss in BMI over ten weeks of follow-up in participants consuming the non-caloric sweetener (-0.9 kg/m^2 ^(-1.5,-0.4), 41 participants). Two trials reported absolute change in weight. One crossover trial was done in type 1 diabetic participants and found no difference in weight loss between groups over four weeks (0.8 kg (-3.3,4.9), ten participants [62]). The other trial in overweight participants [63] found significantly greater weight loss over 10 weeks in the non-caloric sweetener group (-2.6 kg (-3.7,-1.5), 41 participants).

**Table 5:** Weight management, blood glucose and blood lipids: Non-caloric versus Sucrose

Non-caloric sweetener	Population	Timepoint (week)	No of participants	MD (95% CI)
**BMI, kg/m^2^**				
Aspartame	General	4	133	-0.3 (-1.1,0.5)
Mixture^a^	Overweight	10	41	**-0.9 (-1.5,-0.4)**
**Weight, kg**				
Cyclamate	DM1	4	10	0.8 (-3.3,4.9)
Mixture	Overweight	10	41	**-2.6 (-3.7,-1.5)**
**Day Energy Intake, kcal**				
Aspartame	General	4	133	**-283 (-414,-153)**
Mixture	Overweight	10	41	**-491 (-806,-177)**
**HbA1C, %**				
Cyclamate	DM1	4	10	-0.02 (-0.4,0.3)
**HOMA Index**				
Mixture	Overweight	10	41	-0.20 (-0.58,0.18)
**Total Cholesterol, mmol/L**				
Cyclamate	DM1	4	10	-0.34 (-0.87,0.19)
**HDL Cholesterol, mmol/L**				
Cyclamate	DM1	4	10	-0.05 (-0.32,0.22)
**Triglycerides, mmol/L**				
Cyclamate	DM1	4	10	-0.02 (-0.16,0.12)
Mixture	Overweight	10	41	-0.26 (-0.85,0.34)

^a^Aspartame, acesulfame, cyclamate, saccharin

DM1, Type 1 Diabetes Mellitus; DM2, Type 2 Diabetes Mellitus; BMI, Body mass index; HbA1C, Glycated haemoglobin; HOMA, Homeostatic Model Assessment; MD, Mean difference; CI, Confidence interval

Statistically significant results are bolded.

Two trials reported energy intake; both reported a significant effect of non-caloric sweeteners. The 4-week trial in generally healthy participants [61] found a significantly reduced intake of calories in non-caloric sweetener participants (-283 kcal (-414,-153), 133 participants).The trial in overweight participants [63] also found significantly less energy intake (over one day) in the non-caloric sweetener group after ten weeks of follow-up (-491 kcal (-806,-177), 41 participants).

Available trials found no effect of sweetener type on HbA1C (one trial: -0.02% over four weeks (-0.40,0.30), ten participants [62]) or the HOMA index (one trial: -0.20 over ten weeks (-0.58,0.18), forty-one participants [63]). The trial in ten type 1 diabetic participants [62] found no effect on total cholesterol, HDL cholesterol, or triglycerides over the course of four weeks; the other trial in forty-one overweight participants [63] found no effect on triglycerides over the course of ten weeks.

### Saccharide versus saccharide: effects on weight management, blood glucose and blood lipids

Two trials reported change in BMI (Table 6); one comparing honey to sucrose in overweight/obese participants over 4 weeks of follow-up [69]; the other comparing a mixture of isomaltulose and sucrose to sucrose over 12 weeks of follow-up [68] in healthy participants. Neither found a significant difference between sweeteners. One trial compared FOS to glucose [73] (three weeks in twenty diabetic participants) and one trial compared FOS to sucrose [72] (four weeks in twelve healthy participants), respectively. Neither found a difference in absolute weight change. Five other trials done in varying populations (including overweight/obese [69] or healthy populations [64-66,68]) found no differences in change in absolute weight between sweeteners. Two trials reported energy intake (FOS compared with glucose [73] and sucrose [72] respectively, but neither found a significant difference.

**Table 6:** Weight management, blood glucose and blood lipids: Saccharide vs Saccharide

Comparison	Population	Timepoint (week)	No of participants	MD (95% CI)
**BMI, kg/m^2^**				
Honey vs Sucrose	Overweight/Obese	4	55	-0.5 (-3.1,2.1)
Isomaltulose/Sucrose vs Sucrose	General	12	50	-0.04 (-0.4,0.3)
**Weight, kg**				
Fructose vs Glucose	General	6	24	0.1 (-3.4,3.6)
Fructose vs Glucose	General	1	11	-0.4 (-3.1,2.3)
FOS vs Glucose	DM2	3	20	0.2 (-5.2,5.6)
FOS vs Sucrose	General	4	12	1.0 (-2.4,4.4)
Honey vs Sucrose	Overweight/Obese	4	55	-1.5 (-6.9,3.9)
Isomaltulose/Sucrose vs Sucrose	General	12	50	-0.06 (-0.9,0.8)
Sucrose vs Glucose	General	2	10	0.2 (-0.07,0.4)
**Energy Intake, kcal**				
FOS vs Glucose	DM2	3	20	-139 (-399,122)
FOS vs Sucrose	General	4	12	-56 (-156,43)
**HbA1C, %**				
FOS vs Sucrose	DM2	4	10	0.17 (-0.59,0.93)
Isomaltulose/Sucrose vs Sucrose	General	12	50	0.01 (-0.05,0.07)
**HOMA Index**				
Isomaltulose/Sucrose vs Sucrose	General	12	50	**-0.44 (-0.76,-0.12)**
**Total Cholesterol, mmol/L**				
Fructose vs Glucose	General	1	11	0.10 (-0.24,0.44)
FOS vs Glucose	DM2	3	20	0.20 (-0.27,0.67)
FOS vs Sucrose	DM2	4	10	0.15 (-0.24,0.54)
FOS vs Sucrose	General	4	12	**0.31 (0.03,0.59)**
Honey vs Sucrose	Overweight/Obese	4	55	-0.11 (-0.26,0.05)
Isomaltulose/Sucrose vs Sucrose	General	12	50	**-0.10 (-0.17,-0.02)**
Tagatose vs Sucrose	General	4	12	-0.11 (-0.51,0.29)
**LDL Cholesterol, mmol/L**				
Fructose vs Glucose	General	1	11	0 (-0.17,0.17)
FOS vs Sucrose	DM2	4	10	0.13 (-0.21,0.47)
Honey vs Sucrose	Overweight/Obese	4	55	-0.03 (-0.22,0.16)
Isomaltulose/Sucrose vs Sucrose	General	12	50	-0.02 (-0.08,0.04)
Tagatose vs Sucrose	General	4	12	0.09 (-0.26,0.44)
**HDL Cholesterol, mmol/L**				
Fructose vs Glucose	General	1	11	0 (-0.17,0.17)
FOS vs Sucrose	General	4	12	-0.06 (-0.14,0.02)
FOS vs Sucrose	DM2	4	10	0.07 (-0.03,0.17)
Honey vs Sucrose	Overweight/Obese	4	55	0.01 (-0.12,0.14)
Isomaltulose/Sucrose vs Sucrose	General	12	50	-0.02 (-0.05,0.01)
Tagatose vs Sucrose	General	4	12	-0.17 (-0.28,0.06)
**Triglycerides, mmol/L**				
FOS vs Glucose	DM2	3	20	0.12 (-0.30,0.54)
FOS vs Sucrose	General	4	12	0.18 (-0.03,0.39)
FOS vs Sucrose	DM2	4	10	-0.18 (-0.38,0.02)
Honey vs Sucrose	Overweight/Obese	4	55	-0.10 (-0.22,0.02)
Isomaltulose vs Sucrose	General	12	50	**-0.27 (-0.44,-0.10)**

*Aspartame, acesulfame, cyclamate, saccharin

DM1 Type 1 Diabetes Mellitus, DM2 Type 2 Diabetes Mellitus, BMI Body mass index, HbA1C Glycated hemoglobin, HOMA Homeostatic Model Assessment, MD Mean difference, CI Confidence interval

Statistically significant results are bolded.

Two trials (one comparing FOS to sucrose [71] and one comparing isomaltulose/sucrose to sucrose [68]) found no significant effect on HbA1C. However, the latter [68] found a significant decrease in the HOMA index among isomaltulose/sucrose recipients (-0.44 (-0.76,-0.12)).

Seven trials reported change in total cholesterol. The pooled result of two trials [71,72] comparing FOS to sucrose was statistically significant (0.26 mmol/L (0.03,0.48), I^2 ^= 0%, τ^2 ^= 0 (0,0.01)), although this conclusion was based on a total of only twenty-two participants. One trial comparing isomaltulose and sucrose to sucrose (50 healthy participants over 12 weeks) [68] found a significantly smaller increase in total cholesterol for the isomaltulose/sucrose group (-0.10 mmol/L (-0.17,-0.02)). No trials found an effect of sweetener type on LDL cholesterol or HDL cholesterol. The trial comparing isomaltulose and sucrose to sucrose [68] also found a significant effect on triglycerides (-0.27 mmol/L (-0.44,-0.10), 0.11 decrease versus 0.16 mmol/L increase). However, four trials studying other combinations of sweeteners [69,71-73] found no effect of sweetener choice on triglyceride levels.

## Discussion

To our knowledge, this is the first systematic review of randomized trial evidence that examines comparative sweetener effectiveness in diabetic, overweight/obese, and healthy populations. Despite tremendous interest in hypocaloric sweeteners as a potential tool to prevent obesity and its complications, we found little evidence to support their health benefits as compared to caloric alternatives. Based on analyses of two trials, we found that the inclusion of non-caloric sweeteners in the diet resulted in reduced energy intake compared to the caloric (sucrose) groups - approximately 500 kcal/day less over 10 weeks or 250 kcal/day over 4 weeks. The longer of these trials found that those in the non-caloric sweetener group also had a decrease in BMI compared to an increase in BMI in the sucrose group (-0.40 versus 0.50 kg/m^2^, and -1.00 versus 1.60 kg, respectively) [63]. Given that the control group was asked to ingest supplemental calories in addition to their regular *ad lib *diet, a BMI reduction of approximately1 kg/m^2 ^over 10 weeks (or 0·1 kg/m^2^/week) may be overly optimistic. However, even a reduction in BMI of 0.05 kg/m^2^/week would be clinically relevant if sustained for a year or more. The remaining analyses comparing non-caloric and caloric sweeteners were non-significant.

### Main findings

• 53 randomized controlled trials were included - all small and largely short-term (only 13 trials with ≥1 week durations)

• 2-hour blood glucose (mixed evidence, τ^2 ^= 3.66 (95% CI 1.66,7.31): fructose versus sucrose (MD -0.54 mmol/L (-1.06,-0.03)), fructose versus glucose (-0.89 mmol/L (-1.21,-0.59)), fructose versus other sugars (-0.85 mmol/L (-1.47,-0.21)) in non-diabetic participants

• 2-hour blood glucose (direct evidence): fructose versus glucose (-4·81 mmol/L (-6.34,-3.29), I^2 ^= 0%, τ^2 ^= 0 (0,7.47), 5 trials in 52 diabetic participants)

• change in BMI: non-caloric mixture versus sucrose (MD -0.9 kg/m^2 ^[-1.5,-0.4], in 41 overweight participants, over 10 weeks), non-caloric aspartame versus sucrose (-0.3 kg/m^2 ^(-1·1,0·5), 133 healthy participants, over 4 weeks)

• energy intake (over one day): non-caloric aspartame versus sucrose (-283 kcal (-414,-153), 133 healthy participants, over 4 weeks), non-caloric mixture versus sucrose (-491 kcal (-806,-177), 41 overweight participants, over 10 weeks)

• total cholesterol: FOS versus sucrose (0.26 mmol/L (0.03,0.48), I^2 ^= 0%, τ^2 ^= 0 (0,0.01), 2 trials with a total of 12 healthy and 10 type 2 diabetic participants, over 4 weeks)

Head-to-head comparisons between saccharides did not identify any statistically significant differences. The confidence limits of these results either included minimally important differences or the group sizes were too small (< 30) to have good estimates of standard deviation [74]. The one exception was the comparison between sucrose and FOS, which suggested that total cholesterol was reduced to a greater extent with sucrose than with FOS. However, the confidence intervals for this analysis included values that were not clinically relevant (0.03 to 0.59 mmol/L). There was no evidence that HFCS or fructose increased levels of cholesterol relative to other sweeteners.

Although we found that fructose reduced 2-hour blood glucose concentrations by 4.81 mmol/L compared to glucose in diabetic participants, data comparing non-caloric and sugar alcohols to the more commonly used sucrose or HFCS were inconclusive. Contrary to perception and current recommendations, no substantive evidence describing important long-term benefits of hypocaloric sweeteners for diabetic patients were identified. Also, despite popular belief, no high-quality RCT evidence was found indicating that fructose causes or exacerbates hypertriglyceridemia [6].

Although the identified trials were numerous, they were very small and largely short-term. We found 13 trials with participant follow-up longer than 1 week and group sizes ≥ 10: 3 that compared non-caloric sweeteners to sucrose, and 10 that were head-to-head comparisons of saccharides. Ten of 13 trials had a Jadad score of 1 and none adequately concealed treatment assignment prior to assignment. Although blinding the participants would have been impossible in many of the trials due to taste differences between sweeteners [63], the reporting of important design descriptors were largely absent, indicating a substantial risk for bias [12,13]. The longest trial was only 10 weeks - not long enough to determine whether substituting a non-caloric sweetener for a caloric sweetener is sustainable in daily practice. To detect an important reduction in weight over at least one year such as 2.5 kg/m^2 ^(less than 0.05 kg/m^2^/week) in a RCT would require a minimum of 85 participants (assumptions: 25% loss-to-follow-up, α = 0.05, power = 90%, SD = 3 kg/m^2^).

Our network meta-analysis had several limitations: 1) the sugar alcohol and other sugar categories contained multiple sweeteners that are likely to have different blood glucose profiles thereby inducing heterogeneity, 2) power to detect inconsistency is limited by the number of trials included in each test, and 3) the back-calculation method used to detect inconsistency involved multiple tests thereby increasing the false-positive rate. However, we did not detect any inconsistency.

Another limitation was that only three studies restricted the total energy consumed by each participant. Therefore, participants may have supplemented energy lost with non-caloric sweeteners with other food products - sweetened or otherwise. However, it may be argued that this is a strength of the trials - in that they reflect what happens in real world self-management diet practices. Lastly, and perhaps most importantly, all studies were small, thereby underestimating standard deviation and as a result underestimating confidence interval widths and increasing the likelihood of false-positive findings [74]. Despite this, the confidence intervals for many analyses were wide and did not exclude a minimally important difference. Small study bias (or publication bias) may also play a role in our findings concerning longer-term outcomes.

In theory, substituting non-caloric and lower caloric sweeteners for simple sugars should reduce energy intake and thereby the risk of obesity and its consequences. However, there are a number of reasons why increasing use of non-caloric and lower caloric sweeteners might not lead to the expected improvements in energy regulation. First, use of hypocaloric sweeteners might not induce weight loss even in the short term. For example, if reductions in calories due to sweeteners are offset by increases in caloric intake from other sources [75,76], or offset by decreases in caloric expenditure [77,78]. Although our data suggest that non-caloric sweeteners may lead to clinically relevant weight loss through reduced energy consumption, this conclusion was driven by a single trial with a total of 41 participants. Unlike caloric sweeteners (which may partially compensate added calories with reduced energy intake from other sources) [79], non-caloric sweeteners are not known to suppress appetite, and therefore would not reduce the motivation to eat. Furthermore, it has been suggested that the psychobiological signals with non-caloric sweeteners may directly influence physiological regulatory mechanisms and thus further reduce their potential for reducing net energy intake [75,80]. Second, if calorie reduction is not maintained, short-term reductions in weight due to the use of hypocaloric sweeteners might not be sustained. Third, it is possible though speculative that any health benefits due to weight loss from non-caloric sweeteners might be wholly or partially offset by currently unrecognized adverse events due to their use. The lack of data on the long-term benefits of non-caloric sweeteners means that it is currently impossible to determine whether these substances will improve public health.

## Conclusions

In summary, despite the public health importance of obesity, and obesity-related chronic diseases (for example, diabetes); the clear role of excessive caloric intake in these conditions; and the billions of dollars spent on non-caloric sweeteners [4,5], little high-quality clinical research has been done to identify the potential harms and benefits of hypocaloric sweeteners. Since even small reductions (as little as 6%) in body-weight can prevent chronic disease [81,82], hypocaloric sweeteners could play an important role in a wider population health strategy to prevent, reduce and manage obesity-related comorbidities. Eliminating unnecessary added sweeteners from food products (for example, buns, crackers, and processed meats) and substituting sugars with lower calorie sweeteners in foods such as desserts and drinks could significantly improve health. Long-term, high-quality, adequately powered randomized controlled trials are required to confirm this hypothesis by assessing the clinically relevant outcomes reported in this review.

## Abbreviations list

ADA: American Diabetes Association; ADI: Acceptable Daily Intake; BMI: body mass index; CPG: Clinical Practice Guideline; FOS: fructooligosaccharide; HbA1C: glycated haemoglobin; HDL: high density lipoprotein; HFCS: high fructose corn syrup; HOMA: Homeostatic Model Assessment; LDL: low density lipoprotein; MCMC: Markov chain Monte Carlo; MD: mean difference; RCT: randomized controlled trial; SD: standard deviation.

## Competing interests

Dr. Tonelli has received a peer reviewed Canadian Institutes of Health Research Industry Partnership Operating Grant, from Abbott Laboratories. Dr. Field is a scientific advisor to International Life Science Institute North America (non-financial arrangement).

## Authors' contributions

NW conceived the study, did the statistical analyses and wrote the first draft of the manuscript with assistance from MT. All authors contributed to the design, interpretation of results and critical revision of the article for intellectually important content. All authors read and approved the final manuscript.

## Pre-publication history

The pre-publication history for this paper can be accessed here:

http://www.biomedcentral.com/1741-7015/9/123/prepub

## Supplementary Material

Additional file 1 **SweetenerSR contains**. 1) Appendix: Search strategies, 2) Appendix: Study selection criteria box, 3) Appendix Table 1. Mean difference in serum glucose (mmol/L) at 2 hours post-sweetener consumption and overnight fast in non-diabetic participants, 4) Appendix Table 2. Mean difference in serum glucose (mmol/L) at 2 hours post-sweetener consumption and overnight fast in diabetic participants. Click here for file
