# Supplementary material for: A systematic review on the effect of sweeteners on glycemic response and clinically relevant outcomes
Source: BMC Med. 2011 Nov 17;9:123. doi: 10.1186/1741-7015-9-123 (PMC3286380; doi:10.1186/1741-7015-9-123)
Supplement: Additional file 1 — SweetenerSR contains. 1) Appendix: Search strategies, 2) Appendix: Study selection criteria box, 3) Appendix Table 1. Mean difference in serum glucose (mmol/L) at 2 hours post-sweetener consumption and overnight fast in non-diabetic participants, 4) Appendix Table 2. Mean difference in serum glucose (mmol/L) at 2 hours post-sweetener consumption and overnight fast in diabetic participants. [file 1741-7015-9-123-S1.DOC]

Appendix: Search strategies

| Database and Platform | | Search Strategies | Date and Coverage |
| --- | --- | --- | --- |
| MEDLINE (OVID) | | 1. sweetening agents/ or aspartame/ or cyclamates/ or mannitol/ or molasses/ or saccharin/ or sorbitol/ or stevia/ or xylitol/  2. (sweetener* or sweetening agent* or sugar substitute*).ti,ab.  3. (sucralose or splenda or aspartame or nutra sweet or saccharin or stevia or steviol* or acesulfame potassium or sunett or alitame or hernandulcin or isomalt or leucrose or monellin or neotame or sweet protein or thaumatin).ti,ab.  4. Erythritol/  5. (erythritol or lactitol or maltitol or mannitol or sorbitol or xylitol).ti,ab.  6. Honey/  7. (honey or honeys or molass*).ti,ab.  8. (cane sugar or cane juice or sugarcane or rice syrup or barley malt or agave).mp.  9. or/1-8  10. dietary sucrose/ or sucrose/ or sucrose.ti.  11. fructose/ or fructose.ti.  12. lactose/ or lactose.ti.  13. maltose/ or maltose.ti.  14. Glucose/ad, pd [Administration & Dosage, Pharmacology]  15. *Glucose/  16. (glucose adj5 (administ* or ingest* or solution*)).ti,ab.  17. or/10-13  18. 9 or 17  19. (bicycl* or cycling or endurance*).mp.  20. exp mood disorders/ or exp "schizophrenia and disorders with psychotic features"/  21. exp Dementia/  22. cogniti*.ti.  23. exp Cognition/de  24. or/19-23  25. 18 not 24  26. (blood glucose or fasting glucose or plasma glucose).mp.  27. exp hyperglycemia/ or exp hyperinsulinism/ or exp hypoglycemia/  28. (hypoglyc?emi* or hyperglyc?emi* or glyc?emi* or normoglyc?emi*).ti,ab.  29. (hba1c or hemoglobin a1c or a1c).ti,ab.  30. Hemoglobin A, Glycosylated/  31. "body weights and measures"/ or body fat distribution/ or body mass index/ or body weight/ or exp overweight/ or waist circumference/ or skinfold thickness/ or waist-hip ratio/  32. body weight/ or exp body weight changes/  33. (weight gain or weight loss or body fat or body mass or adiposity or obesity).ti,ab.  34. (insulin adj3 (level* or blood)).ti,ab.  35. insulin/ or insulin resistance/  36. insulin.ti.  37. or/26-36  38. 25 and 37  39. animals/  40. humans/  41. 39 not (39 and 40)  42. 38 not 41  43. (Clinical trial or randomized controlled trial or comparative study).pt. or randomi?ed.ti,ab. or placebo.ab. or exp clinical trial/ or randomly.ab. or trial.ti. or double blind*.mp.  44. (review or meta analysis).pt. or (systematic review or meta analysis).ti.  45. (medline or embase or cochrane).ab.  46. or/43-45  47. 42 and 46  48. limit 47 to (addresses or bibliography or biography or case reports or comment or dictionary or directory or editorial or festschrift or guideline or historical article or interview or lectures or legal cases or legislation or letter or news or newspaper article or patient education handout or periodical index or portraits or practice guideline)  49. 47 not 48  50. limit 49 to "all child (0 to 18 years)"  51. limit 49 to "all adult (19 plus years)"  52. 50 not (50 and 51)  53. 49 not 52  54. limit 53 to english language | January 13, 2011  1948-January Week 1 2011 |
| EMBASE (OVID) | | 1. exp Sweetening Agent/  2. (sweetener* or sweetening agent* or sugar substitute*).ti,ab.  3. (sucralose or splenda or aspartame or nutra sweet or saccharin or stevia or stevio* or acesulfame potassium or sunett).ti,ab.  4. (alitame or hernandulcin or isomalt or leucrose or maltitol or monellin or neotame or sorbitol or sweet protein or thaumatin).ti,ab.  5. sugar alcohol/ or erythritol/ or lactitol/ or mannitol/  6. (erythritol or lactitol or maltitol or mannitol or sorbitol or xylitol).ti,ab.  7. Honey/  8. Molasses/  9. (honey or honeys or molass*).ti,ab.  10. (cane sugar or cane juice or sugar?cane or rice syrup or barley malt or agave).mp.  11. Sugar Intake/  12. Sucrose/  13. Lactose/  14. Maltose/  15. (sucrose or lactose or maltose or fructose).ti.  16. or/1-15  17. exp Schizophrenia/  18. exp Dementia/  19. exp mood disorder/ or bipolar disorder/  20. (bicycl* or cycling or endurance*).mp.  21. *cognition/  22. cogniti*.ti.  23. or/17-22  24. Glucose Blood Level/  25. (blood glucose or fasting glucose or plasma glucose).ti,ab.  26. hyperglycemia/ or exp hypoglycemia/  27. (hypogly?cemi* or hyperglyc?emi* or glyc?emi* or normoglyc?emi*).ti,ab.  28. (hba1c or hemoglobin a1c or a1c).ti,ab.  29. Hemoglobin A1c/  30. body weight/ or weight change/ or weight control/ or weight fluctuation/ or weight gain/ or weight reduction/  31. exp Adipose Tissue/  32. obesity/ or abdominal obesity/ or diabetic obesity/ or metabolic syndrome x/ or morbid obesity/  33. body mass/ or waist hip ratio/  34. insulin blood level/  35. (insulin adj3 (level* or blood)).ti,ab.  36. insulin.ti.  37. or/24-36  38. (16 and 37) not 23  39. limit 38 to english language  40. (exp vertebrate/ or animal/ or exp experimental animal/ or nonhuman/ or animal.hw.) not exp human/  41. 39 not 40  42. exp clinical trial/  43. randomi?ed.ti,ab.  44. placebo.ti,ab.  45. randomly.ti,ab.  46. ((single* or double* or triple* or treble*) adj5 (blind* or mask*)).mp.  47. trial.ti.  48. or/42-47  49. 41 and 48  50. (systematic review or meta analysis).mp. [mp=title, abstract, subject headings, heading word, drug trade name, original title, device manufacturer, drug manufacturer name]  51. (medline or embase or cochrane).ab.  52. 50 or 51  53. 41 and 52  54. 49 or 53 | January 13, 2011  1980-2011 Week 1 |
| CENTRAL (Ovid) | | 1. (sweetening agent* or sweetener* or sugar substitute*).mp.  2. (sucrolose or splenda or aspartame or nutra sweet or saccharin or stevia or steviol* or acesulfame potassium or sunett or alitame or hernandulcin or isomalt or leucrose or monellin or neotame or sweet protein or thaumatin).mp.  3. (erythritol or lactitol or maltitol or mannitol or sorbitol or xylitol or honey or honeys or molass* or cane sugar or cane juice or sugarcane or rice syrup or barley malt or agave).mp.  4. (fructose or maltose or sucrose or lactose).mp.  5. 4 or 1 or 3 or 2  6. (blood glucose or fasting glucose or plasma glucose).ti,ab.  7. (hba1c or a1c or hypoglyc?emi* or hyperglyc?emi* or glyc?emi* or normoglyc?emi*).ti,ab.  8. (weight gain or weight loss or weight change* or obesity).ti,ab.  9. (insulin adj3 (level* or blood or resist*)).ti,ab.  10. (glucose tolerance or glucose intolerance).mp.  11. (insulin adj3 (level* or blood or resist*)).mp. or insulin.ti.  12. (weight gain or weight loss or weight change* or obesity).mp.  13. (hba1c or a1c or hypoglyc?emi* or hyperglyc?emi* or glyc?emi* or normoglyc?emi*).mp.  14. (blood glucose or fasting glucose or plasma glucose).mp.  15. or/10-14  16. 15 and 5 | January 13, 2011  4^th^ Quarter 2010 |
| CAB Global Health (Ovid) | 1. (sweetening agent* or sweetener* or sugar substitute*).mp.  2. (sucrolose or splenda or aspartame or nutra sweet or saccharin or stevia or steviol* or acesulfame potassium or sunett or alitame or hernandulcin or isomalt or leucrose or monellin or neotame or sweet protein or thaumatin).mp.  3. (erythritol or lactitol or maltitol or mannitol or sorbitol or xylitol or honey or honeys or molass* or cane sugar or cane juice or sugarcane or rice syrup or barley malt or agave).mp.  4. (fructose or maltose or sucrose or lactose).mp.  5. 4 or 1 or 3 or 2  6. blood sugar/ or glucose tolerance/ or haemoglobin a1/ or hyperglycaemia/ or hypoglycaemia/  7. weight gain/ or weight losses/ or weight reduction/ or obesity/  8. insulin/  9. (blood glucose or fasting glucose or plasma glucose).ti,ab.  10. (hba1c or a1c or hypoglyc?emi* or hyperglyc?emi* or glyc?emi* or normoglyc?emi*).ti,ab.  11. (weight gain or weight loss or weight change* or obesity).ti,ab.  12. (insulin adj3 (level* or blood or resist*)).ti,ab.  13. or/6-12  14. 13 and 5  15. man.od.  16. 15 and 14 | | January 13, 2011  1910-December 2010 |

**Appendix: Study selection criteria box**

| Population:   - healthy, overweight/obese, and/or diabetic - adult (≥16 years)   Interventions (at least two):   - glucose, fructose, sucrose, other saccharides, sugar alcohols, non-caloric sweeteners: aspartame, saccharin, stevioside, sucralose   Outcomes:   - weight change, energy intake, lipids, glycated hemoglobin (A1C), or insulin resistance (at least one week of follow-up) - 2-hour blood (serum or plasma) glucose responses   Study design:   - parallel or crossover randomized controlled trials - ≥10 participants per group - study objective was not exercise performance or memory enhancement |
| --- |

**Appendix Table 1. Mean difference in serum glucose (mmol/L) at 2 hours post-sweetener consumption and overnight fast in non-diabetic participants**

| **Non-**  **caloric**  **0.04** | -0.40  (-1.76,0.97)  τ^2^=3.66  (1.66,7.31) | -0.11  (-0.80,0.67)  consistent | 0.74  (-0.08,1.59) | 0.19  (-0.48,0.90)  consistent | -0.15  (-0.93,0.65) |
| --- | --- | --- | --- | --- | --- |
| - | **Sugar**  **alcohols**  **0.03** | 0.29  (-0.96,1.58) | 1.14  (-0.02,2.33) | 0.60  (-0.61,1.83) | 0.25  (-0.88,1.39)  consistent |
| **-0.40**  **(-0.79,-0.01)**  n=1 | - | **Other**  **sugars**  **0.004** | **0.85**  **(0.21,1.47)** | 0.30  (-0.16,0.75)  consistent | -0.05  (-0.61,0.50)  consistent |
| - | - | - | **Fructose**  **0.92** | **-0.54**  **(-1.06,-0.03)**  consistent | **-0.89**  **(-1.21,-0.59)**  consistent |
| 0.30  (-1.99,2.58)  n=2 I^2^=0  τ^2^=0 | - | 0.21  (-0.70,1.12)  n=6 I^2^=77  τ^2^=0.51  (0.18,0.97) | 0.09  (-0.96,1.15)  n=4 I^2^=0  τ^2^=0  (0,0.07) | **Sucrose/**  **HFCS/**  **Honey**  **0.01** | -0.35  (-0.79,0.07)  consistent |
| - | **0.25**  **(0.02,0.47)**  n=1 | 0.10  (-2.46,2.66)  n=2 I^2^=0  τ^2^=0 | **-0.89**  **(-1.24,-0.53)**  n=18 I^2^=60  τ^2^=0.28 (0.26,0.76) | **-0.28**  **(-0.53,-0.04)**  n=9 I^2^=0  τ^2^=0  (0,0.09) | **Glucose**  **0** |

HFCS high fructose corn syrup

The mixed evidence of the Bayesian network analysis are in the shaded upper triangle and the direct evidence calculated using the REML estimate of τ^2^ are in the lower triangle. Sweeteners are reported in the expected order of efficacy[^17^](#_ENREF_17) (with the exception of other sugars) from the expected lowest to highest 2 hour glucose response, with the estimated probability (or rank) listed in the diagonal. Each table cell contains the mean difference (MD) with the accompanying 95% confidence intervals. In the cells with direct evidence, we also list the number of studies, the I^2^ (percent of heterogeneity due to between-study heterogeneity) and τ^2^ (the between-study variance). Blank cells in the lower triangle indicate that no direct evidence was available. In the cells with mixed evidence, we list whether the mixed evidence was consistent with the available direct evidence. Also, in the first cell of the mixed evidence, we list the single τ^2^ estimate for the mixed evidence. Results are the MD of the expected higher-ranked sweeteners compared to the expected lower-ranked sweeteners (e.g., MD of fructose vs sucrose is 0.09 and is in column 4, row 5 for the direct results, and is -0.54 and is in column 5, row 4 for the network analysis results). MDs less than zero favour the expected higher-ranked sweetener (smaller glucose response). Pooled evidence significant at p<0.05 are presented in bold font. All 8 mixed and direct results are consistent.

**Appendix Table 2. Mean difference in serum glucose (mmol/L) at 2 hours post-sweetener consumption and overnight fast in diabetic participants**

| **Non-**  **caloric** | - | - | - | - | - |
| --- | --- | --- | --- | --- | --- |
| - | **Sugar**  **alcohols**  **0.10** | 1.69  (-2.74,5.90)  τ^2^=224  (0.14,139) | 1.06  (-2.02,4.03) | -1.76  (-4.61,0.96)  consistent | **-3.49**  **(-6.27,-0.74)**  consistent |
| - | - | **Other**  **sugars**  **0.61** | -0.63  (-4.29,3.09) | **-3.48**  **(-6.72,-0.15)**  consistent | **-5.20**  **(-8.65,-1.50)** |
| - | - | - | **Fructose**  **0.29** | **-2.83**  **(-4.40,-1.15)**  consistent | **-4.57**  **(-5.94,-3.05)**  consistent |
| - | 0.41  (-2.44,3.26)  n=1 | **-3.44**  **(-5.31,-1.56)**  n=1 | -2.21  (-4.46,0.04)  n=5 I^2^=0  τ^2^=0  (0,7.27) | **Sucrose/**  **HFCS/**  **Honey**  **0** | **-1.71**  **(-3.02,-0.36)**  consistent |
| - | **-6.19**  **(-9.78,-2.6)**  n=1 | - | **-4.81**  **(-6.34,-3.29)**  n=5 I^2^=0  τ^2^=0  (0,7.47) | -1.13  (-2.76,0.49)  n=6 I^2^=6  τ^2^=0.26  (0.14,9.01) | **Glucose**  **0** |

HFCS high fructose corn syrup

The mixed evidence of the Bayesian network analysis are in the shaded upper triangle and the direct evidence calculated using the REML estimate of τ^2^ are in the lower triangle. Sweeteners are reported in the expected order of efficacy[^17^](#_ENREF_17) (with the exception of other sugars) from the expected lowest to highest 2 hour glucose response, with the estimated probability (or rank) listed in the diagonal. Each table cell contains the mean difference (MD) with the accompanying 95% confidence intervals. In the cells with direct evidence, we also list the number of studies, the I^2^ (percent of heterogeneity due to between-study heterogeneity) and τ^2^ (the between-study variance). Blank cells in the lower triangle indicate that no direct evidence was available. In the cells with mixed evidence, we list whether the mixed evidence was consistent with the available direct evidence. Also, in the first cell of the mixed evidence, we list the single τ^2^ estimate for the mixed evidence. Results are the MD of the expected higher-ranked sweeteners compared to the expected lower-ranked sweeteners (e.g., MD of fructose vs sucrose is -2.21 and is in column 4, row 5 for the direct results, and is -2.83 and is in column 5, row 4 for the network analysis results). MDs less than zero favour the expected higher-ranked sweetener (smaller glucose response). Pooled evidence significant at p<0.05 are presented in bold font. All 6 mixed and direct results are consistent.
